# Factors associated with medical student test anxiety in objective structured clinical examinations: a preliminary study

**DOI:** 10.5116/ijme.5845.caec

**Published:** 2016-12-29

**Authors:** Kyong-Jee Kim

**Affiliations:** 1 Department of Medical Education, School of Medicine, Dongguk University, Korea

**Keywords:** Test anxiety, objective structured clinical examinations (OSCE), medical students, Korea

## Abstract

**Objectives:**

To investigate attributes of medical students associated with their test anxiety on Objective Structured Clinical Examinations (OSCEs).

**Methods:**

A cross-sectional study using a self-administered questionnaire was conducted of all Year 3 and 4 students at a private medical school in South Korea in 2014. This 53-item questionnaire consisted of factors pertaining to test anxiety on the OSCE identified from a review of relevant literature, which included students’ motivational beliefs and achievement emotions, perceived values of the OSCE, and attitude and orientation towards patients. Participants’ test anxiety levels were measured using the Korean Achievement Emotions Questionnaire. Participants rated their responses using a five-point Likert-type scale. Univariate analysis was performed to examine relationships between the variables.

**Results:**

A total of 94 students completed the questionnaire (a 93% response rate). No differences in the participants’ test anxiety scores were observed across genders, entry-levels, or years in medical school. Participants’ test anxiety on the OSCE showed moderate association with their class-related achievement emotions (i.e., anxiety and boredom), where r = 0.46 and 0.32, p < 0.01, respectively, and weak negative associations with their patient-centeredness (r = -0.21, p < 0.05) and with their perceived values of the OSCE (r = -0.21, p < 0.05).

**Conclusions:**

This study found some non-cognitive factors related to medical students’ test anxiety on the OSCE. These findings have implications for developing effective educational interventions for helping students cope with such a stress by enhancing our understanding of the various factors that influence their test anxiety in OSCEs.

## Introduction

Research indicates that student performance on clinical performance examinations, such as the Objective Standardized Clinical Examination (OSCE), is mediated by the individual’s non-cognitive traits, such as his or her perceptions of anxiety, self-confidence, and preparedness, in addition to his or her knowledge and skills.^1^^,^^2^ Accordingly, medical educators need to understand various non-cognitive factors that influence student performance on the OSCE. In particular, the literature indicates that the OSCE is a stressful assessment method for students, which evokes more anxiety than other examinations.^2^^,^^3^ This is due, in part, to the fact that the format of the OSCE is unfamiliar to students, thus it can cause anxiety, particularly in summative assessment settings.^4^ Still, previous studies have shown medical students are not adequately prepared to cope with such factors that influence their anxiety in OSCEs.^2^ Thus, this study intends to investigate non-cognitive factors that influence medical students’ test anxiety on OSCEs.

Several non-cognitive factors that influence student performance have been reported in the literature. Research suggests that students’ motivational beliefs and achievement emotions influence their learning and performance.^5^ Motivation influences student learning and performance^6^ and is also a predictor for psychological well-being, such as distress and burnout.^7^ Despite a wealth of research on student motivation in medical education, research linking student motivation to clinical performance is lacking. Still, theories suggest there are individual differences in perception regarding the importance of success or failure on a cognitive task (task valence) and individual self-beliefs on the ability to perform the task (expectancy beliefs), which potentially impact student anxiety and performance in achievement situations.^8^

Furthermore, theories suggest achievement emotions, which pertain to emotions on achievement activities or achievement outcomes (e.g., enjoyment, boredom, or anxiety), influence students’ motivation, learning, and performance.^9^^,^^10^ Although achievement emotions have increasingly garnered attention, there is scant empirical evidence in medical education research. In particular, test anxiety, an achievement emotion known to influence students’ academic performance, can occur with the OSCE and for some students it may negatively impact their performance on it. Yet, past studies of test anxiety has largely been on academic performance, and little is known about factors that influence medical students’ anxiety in the test settings which involve clinical performance such as in OSCEs.

In particular, as interaction of examinees with simulated patients is key to successful performance on the OSCE, it is speculated that their orientations and attitudes towards patients influence their emotions associated with the OSCE. Research shows that physicians’ practice attitudes towards patients are associated with patient satisfaction,^11^^,^^12^ and Hur and colleagues^13^ reported that exposure of medical students to the OSCE influences their attitudes in patient-physician relationships. However, empirical evidence is lacking on the relationship between medical students’ attitudes towards patients and their performance or test anxiety on the OSCE.

This study identified various non-cognitive factors that are speculated to influence medical students’ test anxiety on OSCEs from the review of relevant literature, which were motivational beliefs and achievement, perceived values of the OSCE, and their orientations and attitudes towards patients. Based upon this conceptual framework, this study aimed to investigate associations of these non-cognitive attributes with medical students’ test anxiety on OSCEs.

## Methods

### Study participant and setting

A cross-sectional study was conducted of a sample (n = 101) of Year 3 and 4 medical students at Dongguk University medical school (DUMS), a private medical school in Korea. All 3rd and 4th year students in the basic medical program at DUMS were invited to participate in the study. The participants were in clinical rotations following two years of pre-clinical education in the four-year medical program. Approximately one-third of the students were undergraduate-entry, and two-thirds were graduate-entry students. Students of both entry-levels were in the same four-year medical curriculum, in which undergraduate-entry students had taken a two-year premedical program that preceded the medical program.

The questionnaires were completed by 94 out of 101 students, resulting in a 93% response rate; 45% (n = 42) of the participants were female and 55% (n = 52) were male; ages ranging from 21 to 31 years (M = 26.8, SD = 3.62), with 36% (n = 34) undergraduate-entry and 64% (n = 60) graduate-entry students.

Students were exposed to OSCEs in their clinical years in the manner that they would be assessed in the national licensing exams. In this testing format, students are given a total of 12 stations in which they perform patient encounters, including history-taking, and physical examinations, and clinical procedures. Clinical procedures are assessed by medical faculty, and patient encounters are evaluated by standardized patients. Year 1 and 2 medical students, who were in the pre-clinical years, had no experience with the OSCE and were therefore excluded from this study.

### Instrument and procedures

Data were collected in April 2014, using a self-administered questionnaire, which was designed based on literature. The questionnaire consists of 53 items, including participant demographics (6 items), participants’ test anxiety levels on the OSCE (6 items), their motivational beliefs and achievement emotions (21 items), their orientation and attitudes towards patients (18 items), and their perceived values of the OSCE (2 items).

The Korean Achievement Emotions Questionnaire (K-AEQ) was used for measurement of participants’ test anxiety with the OSCE. The questionnaire was originally developed by Pekrun and colleagues,^14^ and its Korean version was developed and validated by Do and colleagues.^15^ The K-AEQ consisted of 80 items measuring nine types of emotion in three different contexts – class-related, learning-related, and test-related settings. Six items on anxiety in the test-related setting were adapted for this study. Cronbach’s alpha for these six items was 0.88.

Participants’ motivational beliefs and achievement emotions were measured using a questionnaire developed by Artino and colleagues.^5^ This instrument comprises 5 sub-scales, two pertaining to motivational beliefs (task value and self-efficacy; 10 items) and three regarding class-related achievement emotions (enjoyment, anxiety, and boredom; 11 items). This instrument was translated by the author and had been previously pilot tested with 40 medical students to evaluate the clarity of the items. Cronbach’s alpha values of these five sub-scales ranged between 0.79 and 0.87.

The Korean version of the Patient-Practitioner Orientation Scale (PPOS) was used for measurement of student attitudes and orientation towards patients. PPOS measures the extent to which the respondent is patient-centered in his/her practice attitudes.^12^ PPOS consists of two sub-scales, sharing and caring, with a total of 18 items. The Korean version of PPOS, which was developed and validated by Sohn and colleagues^16^ was used for this study. Cronbach’s alpha values of the two sub-scales of the PPOS in this study were 0.83 and 0.89.

Participants’ perceptions of values of the OSCE were measured using two items, “it is important for me to do well on the OSCE to become a competent physician” and “OSCE is an appropriate method to assess clinical competence.” The Cronbach’s alpha value of these two items was 0.85.

Participants rated their responses on a five-point Likert-type scale, where 1 = “strongly disagree” and 5 = “strongly agree.” In addition, participants’ cumulative GPAs and OSCE scores were obtained to investigate the relationship between their academic and clinical performance and their test anxiety on the OSCE.

The questionnaire was self-administered in a paper-and-pencil format. The students filled in the questionnaire at the end of their clerkship sessions, where they met either in small groups or with the whole class. The current study fell under the general exemption from our institutional review board for educational outcomes data; therefore IRB approval was not requested. Participation was voluntary and consent was implied with the return of the survey. To protect student anonymity and ensure voluntary participation, each student completed the questionnaire privately and anonymously and returned it in a sealed envelope.

### Statistical analysis

Descriptive statistics was performed of OSCE scores obtained and these scores were analyzed using independent t-test to compare student performance in OSCE across different backgrounds. Furthermore, correlation analysis was performed to investigate the associations between the variables being studied and independent t-test was performed for comparison of participants’ test anxiety on the OSCE across different backgrounds. IBM-SPSS version 22 for Windows was used and the significance level was 0.05 for the statistical analysis.

## Results

The mean score of the participants’ test anxiety on the OSCE was 3.38 (SD = 0.64). No differences in participants’ test anxiety scores were observed across genders (t = 1.05, p = 2.99), or across entry-levels (t = 0.86, p = 0.39), or across years in medical school (t = 0.39, p = 0.70).

Correlations between participants’ test anxiety on the OSCE and related variables are shown in Table 1. There were no significant associations between participants’ test anxiety scores on the OSCE and their motivational beliefs, yet these showed moderate association with their achievement emotions (i.e., anxiety and boredom), where r = 0.46 and 0.32, p < 0.01, respectively. In addition, participants’ test anxiety scores on the OSCE showed weak negative associations with their total PPOS scores (r = -0.21, p < 0.05) and with their perceived values of the OSCE (r = -0.21, p < 0.05). Still, participants’ test anxiety with the OSCE did not show significant association with their OSCE scores obtained (r = 0.09, p = 0.40), or with their accumulative GPAs (r = 0.09, p = 0.41).

**Table 1:** Pearson Correlations among students’ test anxiety on the OSCE and the study variables (N = 94)

Factors		Test anxiety on OSCE (Pearson’s r)
Motivational beliefs		
	Self-efficacy	0.084
	Task value	0.012
Achievement emotions (class-related)		
	Enjoyment	-0.016
	Anxiety	0.465**
	Boredom	0.317**
Patient-centeredness (PPOS^†^ scores)		
	Sharing	0.137
	Caring	-0.250*
	Total	-0.208*
Perceived values of OSCE		-0.208*
Outcome measures		
	OSCE scores obtained	0.089
	Cumulative GPAs	0.086

*p > 0.05, **p > 0.01

†Patient-Practitioner Orientation Scale

## Discussion

This study found some factors related to medical students’ test anxiety on the OSCE. Our findings show that medical students who exhibit more negative achievement emotions related to classes may likely feel more anxious about the OSCE. These achievement emotions were more strongly associated with their test anxiety on OSCEs than any other factors being studied.

In addition to class-related achievement emotions, this study shows that medical students’ perceived values of the OSCE are associated with their test anxiety on the OSCE. This finding is in line with those of previous studies and educational theories indicating an association between task values and test anxiety in academic performance settings.^8^ Our finding suggests such a relationship also applies to the clinical performance environment. Attaching a high value to the task is positive in that it leads to more engagement in learning, but it can also negatively impact student performance when it induces high levels of test anxiety.^17^ As training in the use of effective learning strategies and test-taking skills can help reduce the degree of test anxiety,^17^such training is warranted for students with high levels of test anxiety.

Furthermore, this study indicates that student perceptions of patient-physician relationship are associated with their anxiety on the OSCE. It can be interpreted that those with higher PPOS scores more likely have a patient-oriented style of interaction with patients, and those with such an orientation likely feel more comfortable and therefore less anxious about interacting with the patients in the OSCE setting. In general, medical students have no opportunity to practice OSCEs other than in high-stakes examinations and mock OSCEs may reduce anxiety associated with OSCEs.^18^ Thus, mock OSCEs may help students lessen their anxiety caused by the unfamiliarity with the test format.

Still, this study shows no relationship between medical students’ test anxiety and their performance on the OSCE. Furthermore, our finding shows that medical students’ test anxiety on the OSCE is not associated with their academic performance. This finding confirms our speculation that academic performance is a weak predictor for medical students’ test anxiety on the OSCE and reaffirms the need for a better understanding of individual differences and personal factors linked to test anxiety on the OSCE.

Limitations of the study should be acknowledged. First, this was a preliminary study using a relatively small sample from one institution. Therefore, future research with a larger sample from multiple institutions is warranted to enhance the generalizability of this study. Furthermore, a multivariate analysis is recommended for a more comprehensive understanding of factors that influence students’ test anxiety in OSCEs and to investigate causal relationships among these factors. Second, this study used a self-administered questionnaire method, where issues of response bias such as social desirability^19^ can arise. Additional studies using a qualitative research method is recommended to enhance validity of this study by using multiple data sources and to gain more in-depth understanding of the impact of test anxiety on student performance in OSCEs.

## Conclusions

This study was a preliminary investigation into factors associated with medical students’ test anxiety on the OSCE. This study sheds light on attributes of medical students who likely feel more anxious about the OSCE, which may hinder their performance. The findings from this study have implications for developing effective educational interventions for helping students cope with such a stress by enhancing our understanding of the various factors that influence their test anxiety in OSCEs. This study warrants further study for a more comprehensive understanding of factors influencing medical students’ test anxiety on the OSCE. Such studies would help us advance our knowledge of individual differences in factors influencing medical students’ test anxiety and in its impact on their performance in OSCEs.

### Conflict of Interest

The authors declare that they have no conflict of interest.
